# Innovative public-private partnerships to maximize the delivery of anti-malarial medicines: lessons learned from the ASAQ Winthrop experience

**DOI:** 10.1186/1475-2875-10-143

**Published:** 2011-05-23

**Authors:** François Bompart, Jean-René Kiechel, Robert Sebbag, Bernard Pecoul

**Affiliations:** 1 Sanofi-Aventis, Access to Medicines Department, 74- 82 Avenue Raspail 94255 Gentilly Cedex, France; 2 Drugs for Neglected Diseases initiative, 15 Chemin Louis-Dunant,1202 Geneva, Switzerland

## Abstract

**Background:**

This case study describes how a public-private partnership initiated to develop a new anti-malarial combination, ASAQ Winthrop, has evolved over time to address issues posed by its effective deployment in the field.

**Case description:**

In 2002, DND*i *created the FACT project to develop two fixed-dose combinations, artesunate-amodiaquine and artesunate-mefloquine, to meet the WHO anti-malarial treatment recommendations and international regulatory agencies approval standards. In 2002, Sanofi-aventis had started a development programme for a fixed-dose combination of artesunate and amodiaquine, to replace its co-blister combination. DND*i *and sanofi-aventis joined forces in 2004, with the objective of developing within the shortest possible time frame a non-patented, affordable and easy to use fixed-dose combination of artesunate and amodiaquine adapted to the needs of patients, in particular, those of children. The partners developed Coarsucam^®^/Artesunate Amodiaquine Winthrop^® ^("ASAQ Winthrop") which was prequalified by the WHO in 2008. Additional partnerships have since been established by DND*i *and sanofi-aventis to ensure: 1) the adoption of this new medicine by malaria-endemic countries, 2) its appropriate usage through a broad range of information tools, and 3) the monitoring of its safety and efficacy in the field through an innovative Risk Management Plan.

**Discussion and evaluation:**

The partnership between DND*i *and sanofi-aventis has enabled the development and pre-qualification of ASAQ Winthrop in a short timeframe. As a result of the multiple collaborations established by the two partners, as of late 2010, ASAQ Winthrop was registered in 30 sub-Saharan African countries and in India, with over 80 million treatments distributed in 21 countries. To date, 10 clinical studies, involving 3432 patients with ASAQ Winthrop were completed to document efficacy and safety issues identified in the Risk Management Plan.

**Conclusions:**

The speed at which ASAQ Winthrop was adopted in the field shows that this drug fits the needs of patients and health authorities. It also demonstrates the power of partnerships that combine different sets of strengths and skills, and that evolve to include additional actors to meet new global health challenges for poverty-related diseases.

## Background

Due to the high efficacy of artesunate-based combination therapy (ACT) in the treatment of uncomplicated malaria in Africa and Asia, a technical consultation organized by the WHO in 2001 recommended their use to provide effective treatment against malaria and to slow down the spread of drug resistance. In 2006, WHO further stated its preference for ACT in fixed-dose combinations to overcome the issues related with non-fixed dose options [1]. The fixed dose combination had been identified by experts assembled in 2003 by the WHO as highly desirable in terms of adherence and ease of treatment, containment of resistance, reduction of diversions and possibly reduction of costs [2].

A partnership was initiated in 2004 between the Drugs for Neglected Diseases *initiative *(DND*i*) and sanofi-aventis to develop together a fixed-dose combination of artesunate and amodiaquine, one of the forms of ACT recommended by the WHO, which at that time only existed as a non-fixed combination of the two drugs. This case study aims at retracing the main features of this partnership, and describes how it was progressively enlarged to include new partners to maximize the launch and distribution of the new medicine. The lessons from this experience may help in the development of other need-driven drugs.

## Case description

### The FACT project: 2002-2004

In 2002, under the auspices of the DND*i *Working Group, which led to the creation of the DND*i *foundation in 2003, the Fixed-Dose Artesunate-based Combination Therapies (FACT) Project was initiated. Its aim was to develop two fixed-dose combinations, namely artesunate-amodiaquine and artesunate-mefloquine, in the shortest possible timeframe to meet the WHO treatment recommendations and international quality standards. The FACT project consisted of DNDi, Farmanguinhos/Fiocruz (Brazil), Tropival of the Bordeaux II Victor-Segalen University (France), Oxford University (UK), Universiti Sains (Malaysia), Mahidol University (Thailand), the Special Programme for Research and Training in Tropical Diseases WHO/TDR (Switzerland), and the Centre National de Recherche et de Formation sur le Paludisme (Burkina Faso). In line with DND*i*'s mission of collaboration based on relative strengths, the FACT project capitalized on the skills and know-how of a broad range of partners in both developing and developed countries. Initially, the FACT project was financially supported by the European Union (INCO-Dev programme) and Médecins Sans Frontières, then by government agencies from France, the Netherlands, Spain and the UK. At the end of 2004, the formulation of the "bilayer" tablet had been developed with good stability characteristics and a packaging that guaranteed stability in tropical field conditions [3,4]. A first scale-up to pre-industrial stage had occurred at Rottendorf Pharma (Germany). Clinical studies were ongoing in healthy volunteers in Malaysia and in patients in a comparative study in Burkina Faso [5]

Meanwhile, in 2004, sanofi-aventis had registered and launched a co-blistered, non-fixed form of artesunate and amodiaquine (Arsucam®) in sub-Saharan Africa. Recognizing the advantages of fixed-dose combinations to improve patient compliance, dosing accuracy and avoiding the risks related with monotherapy, the company had also initiated, in 2002, a programme to develop a fixed-dose combination of artesunate and amodiaquine.

### The sanofi-aventis - DND*i *development partnership: 2004-2008

In December 2004, DND*i *and sanofi-aventis decided to join forces to form a public-private partnership with the objective of developing a non-patented fixed-dose combination of artesunate and amodiaquine that would be made available at prices lower than those available at the time, i.e. less than US$1 for an adult treatment and less than US$0.50 for a child's treatment in the non-profit public sector. Sanofi-aventis did not seek any patent protection. This decision was made to be consistent with DND*i*'s intellectual property policy to develop drugs as public goods when possible, and with both partners' determination to make the new medicine widely available to those patients in greatest need. Sanofi-aventis agreed to pay DND*i *3% of the net private sector turnover over a period of seven years. DND*i *decided to use this payment to support the Risk Management Plan, which is described later in this paper.

DND*i*'s key contribution at the initial stage of the partnership was brought by the University Victor-Segalen of Bordeaux and then by Ellipse Pharma (France), which developed the "bi-layer" formulation with the objective of keeping separated the artesunate and the amodiaquine components, and defined the key features of the manufacturing process of the fixed-dose combination. The process allowed for a reduction in the size and number of tablets, as well product stability in tropical climates [3,6].

Once collaboration was started, regular joint projects teams between sanofi-aventis and DND*i *enabled making the most of each partner's skills and resources, and a steering committee provided arbitrage in case of disagreement. Project teams composition evolved over time as required by the various steps of the development (regulatory, pre-clinical, clinical, industrial, etc.). Meetings between the two parties were jointly managed by one representative of each partner. The roles and responsibilities of each partner, and, therefore, the rules for decision-making, were laid out early on. DND*i *was responsible for the actual formulation of ASAQ Winthrop, early Phase I data, as well as for one pivotal comparative study. Sanofi-aventis was in charge of the manufacturing process at industrial scale, managing the second pivotal clinical study, building the registration file, ensuring registration in endemic countries, obtaining WHO pre-qualification and launch and marketing of the new drug. Decisions which could not be reached by consensus between the two parties were discussed at steering committee meetings which occurred approximately four times a year. When consensus could still not be reached, the final decision was made by the party responsible for carrying out the task under consideration. Collaborative work between the two partners led to the following outcomes:

#### Adapted profile and dosage

The development of the ASAQ Winthrop tablets strengths was focused towards optimizing safe and effective drug levels across all age groups, with a specific emphasis on the needs of children who are the first victims of malaria. The formulation of ASAQ Winthrop enables the drug to be dissolved in water, making it easier to administer to infants and small children. In most endemic countries, dosing recommendations based on age are simpler to use in the field than those based on bodyweight. DND*i *was of the opinion that the age-based dosing was more practical in developing countries. Sanofi-aventis, however, was in favour of developing a weight-based formulation in keeping with major regulatory agencies regulations. Both partners came to consensus thanks to an innovative body of work that modeled actual weight-for-age data from malaria endemic African countries [7]. This dataset, referred to as the malaria weighted anthropometric reference (MWAR) dataset, allowed the design of practical weight and age-based regimens to maximize the proportions of patients receiving appropriate drug doses, while avoiding the risk of over- and under-dosing artesunate and amodiaquine. The strategy of dose accuracy provided a definition of acceptable dosing ranges for both drugs. The dosing ranges for artesunate and amodiaquine were:

- artesunate: 2 to 10 mg/kg (5 fold), reflecting its tolerability and, therefore, wide

- therapeutic index.

- amodiaquine: 7.5 to 15 mg/kg (2 fold). There was less flexibility with amodiaquine, therefore, amodiaquine determined the final dosing and age categories.

In addition, two conventions were adopted:

- select age groups with an approximate doubling in median bodyweight, and

- double the drug dose per age category.

**Figure 1:**
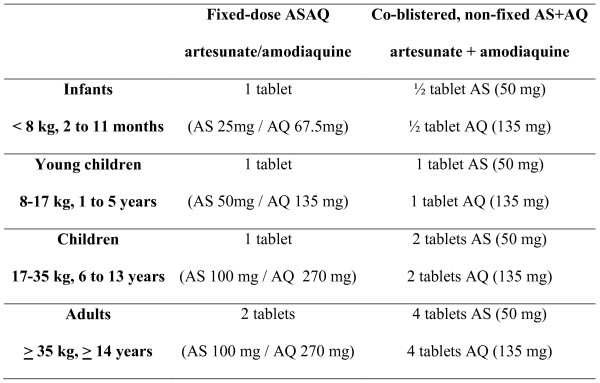
**ASAQ Winthrop and non-fixed combination dosing regimens**. Simplified dosing regimen with fixed-dose ASAQ. The bi-layer formulation allows for AS and AQ to be taken together, in correct proportions, with fewer tablets as compared with the co-blistered non-fixed dose options.

These conventions led to selecting 4 dosage forms based on age and weight ranges, for infants (4.5 to 8 kg, 2 to 11 months), toddlers (9-17 kg, 1 to 5 years), children (18-35 kg, 6 to 13 years) and adults (> 36 kg, 14 years and above). Figure 1 shows the dosing regimen of ASAQ Winthrop, compared with that of the non-fixed combination.

#### Preparation of the registration dossier

Between 2004 and 2007, a joint project team was set up between sanofi-aventis and DND*i *to cover all tasks required to license ASAQ Winthrop: industrial pharmaceutical development (i.e. development of processes required to reach production on an industrial scale at the Maphar-sanofi-aventis plant in Casablanca, Morocco); pre-clinical pharmaco-toxicology studies to document the safety profile of artesunate plus amodiaquine in animals; clinical (from Phase I studies to document the drug's pharmacokinetics, to Phase III well-controlled clinical studies to document its safety and efficacy in malaria patients); regulatory (preparation of registration files for Morocco, sub-Saharan malaria endemic countries and WHO pre-qualification process), as well as marketing and medical activities to prepare the drug's launch and marketing in endemic countries and design post-launch clinical studies to monitor its safety and efficacy in the field.

The registration dossier included clinical data on 1,003 patients treated with ASAQ Winthrop from two randomized controlled studies: one managed by DNDi in Burkina Faso comparing ASAQ Winthrop to a non-fixed combination of artesunate and amodiaquine [5], and another study managed by sanofi-aventis, set up in Senegal, Mali, Cameroon and Madagascar, comparing ASAQ Winthrop to artemether-lumefantrine [8]. In addition, clinical data on over 6,000 patients treated with non-fixed combination of artesunate and amodiaquine were provided.

#### Registration of Coarsucam/ASAQ Winthrop

Many discussions were held over the best way to register ASAQ Winthrop to make it available as quickly as possible in endemic countries, while demonstrating its adherence to high-quality standards. It was decided to seek initial registration from Morocco, the country where the drug is manufactured, to enable registration in endemic countries, but to also apply for WHO prequalification as further evidence of ASAQ Winthrop's quality standards, and to enable purchasing by international agencies, The Casablanca production plant was chosen in line with sanofi-aventis' commitment to develop its existing industrial assets in developing and emerging countries. A major financial investment was made to ensure appropriate production capacity level, as well as adherence of the plant to Good Manufacturing Practice standards. To allow for differential pricing between the public and private markets, two brand names were selected: Coarsucam^®^, for private markets (1 blister per box), and Artesunate Amodiaquine Winthrop^® ^for public markets (25 blisters per box). Winthrop is the name of sanofi-aventis generic range of medicines. The shortened name "ASAQ Winthrop" in this article refers to both brand names and is used to highlight the fact that this product is a specific fixed-dose combination of artesunate and amodiaquine. The registration dossier was submitted in December 2005 and approval was granted in Morocco on February 1, 2007, making ASAQ Winthrop the first new anti-malarial treatment developed and delivered by a public-private partnership [6]. Following this, registration applications were submitted in 30 malaria-endemic African countries and approval were obtained between November 2006 (in Benin) and December 2010. The decisive step which enabled rapid exportation to other African countries was registration in February 2007 in Morocco, the country of manufacturing of the medicine, Sanofi-aventis submitted a dossier to the WHO pre-qualification process in February 2007. Following review of the submitted data, and two Good Manufacturing Practice inspections of the manufacturing plant, the WHO granted a "pre-qualified" status in October 2008. This status opened the door to purchases of ASAQ Winthrop by malaria-endemic countries funded by international organizations resulting in 6 million treatments in 2008 to 25 million in 2009 and over 45 million in 2010. In keeping with the principles of sanofi-aventis' Access to Medicines approach, ASAQ Winthrop is made available through a tiered-pricing policy, that includes "no profit-no loss" prices for ASAQ Winthrop in the public sector (governments, non-profit NGOs, etc.), while the same drug is sold in the private sector under a different brand name (Coarsucam^®^), at market prices that include profit margins. Specifically, ASAQ Winthrop is available in the public sector at prices below $1 for adults and $0.50 for children, while Coarsucam^® ^is sold for approximately $2-$3 to wholesalers for the private markets. The profit margins made though sales in the private sector ensure that the mechanisms that enable very low "no profit-no loss" prices in the public sector can be sustained over the long-term.

#### The ASAQ Winthrop Risk Management Plan

Early in their collaboration, sanofi-aventis and DND*i *wanted to ensure that appropriate post-marketing data was available as quickly as possible on ASAQ Winthrop safety and effectiveness in the field. This was deemed necessary because of the relatively poor image of amodiaquine safety, in spite of its re-installment in the 1990s as a safe and effective drug for the treatment of malaria [9]. Also, because multiple associations of artesunate and amodiaquine, with different drugs ratios and quality standards, have been used in the past, it was felt crucial to document the specific safety and efficacy profile of ASAQ Winthrop. Further no pharmacovigilance data could be expected from industrialized countries and existing pharmacovigilance systems in sub-Saharan Africa were not sufficiently operational to reliably document the safety profile of this drug. The "ASAQ Winthrop deployment monitoring plan" was, therefore, designed to provide quality efficacy and safety data through a variety of proactive studies, each providing different types of data. These studies range from randomized, comparative, clinical trials in limited number of patients treated under well-controlled conditions (who would be extensively followed-up during each of their treated malaria episodes) to large-scale studies assessing the drug's safety in "real-life" conditions. These studies are performed in several countries of West and East Africa, with various malaria transmission patterns, to provide a comprehensive picture on the safety and effectiveness of ASAQ Winthrop. This programme includes the following key studies, which are at various stages of completion:

- Several randomized controlled studies in sub-Saharan African countries that provide safety and efficacy data on ASAQ Winthrop, compared with other forms of ACT, usually in the treatment of single malaria episodes in patients with documented malaria.

- Two cohort studies in Senegal and in Uganda: in each study, cohorts of 200 patients were randomly assigned to receive either ASAQ Winthrop or artemether-lumefantrine for each malaria attack occurring during the two-year study period. The Uganda study focuses on children less than five years of age. The Senegal study includes both adults and children.

- A Phase IV field programme to assess ASAQ Winthrop "real life" safety and effectiveness over two years in a Côte d'Ivoire health district that uses ASAQ Winthrop as first-line treatment, through two approaches. First, an active monitoring of clinical tolerability and compliance for all malaria patients attending district health centres for treatment, performed by community health care workers who visit treated patients at home to collect information. In this study, patients diagnosed with malaria are prescribed ASAQ Winthrop to take at home under non-supervised conditions as is standard medical practice currently in Côte d'Ivoire. Blood smears are collected to enable post-treatment laboratory diagnosis of malaria, to identify which patients definitely had malaria. This approach will enable getting a "real-life" view of the drug's safety and efficacy in patients treated after clinical diagnosis as frequently happens in Africa, and to assess possible differences between patients with and without biologically-proven malaria. The World Malaria Report 2009 [10] and the Guidelines for the Treatment of Malaria 2010 of the WHO [11] recommend prompt parasitological confirmation by microscopy or with Rapid Diagnostic Tests for all patients with suspected malaria before treatment is started. When Côte d'Ivoire implements these recommendations, the study procedures will be changed accordingly. The second part of the programme consists in a clinical safety and efficacy study, at baseline and after two to three years of implementation, to assess the evolution of malaria parasites susceptibility to the artesunate-amodiaquine combination under well-controlled conditions.

As more data on ASAQ Winthrop, generated through studies managed by either one of the two partners or by other institutions become available, they will be pooled into a common database and analysed by an academic investigation team to provide comprehensive information on the drug's efficacy and safety.

The WHO Department of Medicines Policy and Standards expressed a strong interest in this programme, and requested a formalized Risk Management Plan (RMP). This was done in 2009, and a series of documents was submitted following the format required by European Medicines Agency for RMP. Table 1 shows the list of risks and missing information that will be documented through the RMP. This is the first RMP ever submitted to the WHO and is based almost exclusively on data collected in Africa. It is expected that the experience drawn from this initiative will help design RMPs for future new anti-malarials that will be launched in developing countries with relatively limited sets of safety and efficacy data.

**Table 1:** ASAQ Winthrop Risk Management Plan: issues to be documented

**1.**	**Identified risks: to be minimized with specific information**
	• Intake during first trimester of pregnancy
	• Allergy
**2**.	**Potential risks: to be quantified in large-scale studies**
	• Hepatotoxicity
	• Neutropenia/agranulocytosis
	• Somnolence
	• Audiometric dysfunction
	• Extra-pyramidal symptoms
	• Decreased efficacy (parasite resistance)
**3**.	**Missing information: to be documented in new studies**
	• Safety of repeated administrations
	• Specific populations (HIV/AIDS patients...)
	• Second and third trimester of pregnancy
	• Safety profile in non parasitaemic patients
	• Drug interactions & Interactions with traditional drugs and remedies
	• Efficacy in species other than *P. falciparum*

In 2009, the Medicines for Malaria Venture (MMV) joined the sanofi-aventis/DND*i *partnership to set up the Côte d'Ivoire field programme, the most ambitious component of the RMP, and is funding most of the costs thereof. This collaboration was seen by MMV as a way to develop and test methodologies that could be relevant to design RMP for future anti-malarials.

#### Partnering with malaria-endemic countries

The partnership that led to the development and registration of ASAQ Winthrop had to evolve to ensure the new drug's successful launch and adoption by endemic countries. First among the many partners needed at this crucial stage are the endemic countries' National Malaria Control Programmes (NMCP) that are charged with the design and implementation of national action plans to control malaria. In working to facilitate the implementation and availability of ASAQ Winthrop, both sanofi-aventis and DND*i *have engaged in collaborations with a number of partners, including experts, national malaria programmes, WHO and other international organizations, research institutes, funding agencies, and NGOs. DND*i *has, for instance, coordinated a clinical study with its founding partner, the Indian Council of Medical Research, to facilitate the adoption of a new anti-malarial policy in India; this study allowed registration of ASAQ Winthrop in India. DND*i *has also convened an independent panel of experts, the FACT Implementation Advisory Group, to provide independent advice and critical guidance about issues related to implementation and rational use of ASAQ Winthrop, ensuring equitable access.

#### Information and educational tools

Sanofi-aventis has focused its efforts on supporting the introduction of the new treatment in public health systems through a series of information tools to assist with the change in prescription and dispensing habits. In partnership with African NMCP and malaria experts, a variety of information and educational tools were designed, not only about the rationale and instructions on how to use a fixed-dose ACT, but more broadly about the comprehensive management of the disease, that includes malaria prevention, diagnosis and treatment. Specific supports are designed for each "constituency" involved in the fight against malaria, such as textbooks, slide kits, CDs, educational flipcharts, games, etc. Information for physicians was focused on malaria diagnosis and treatment, while those aimed at communities focused primarily on malaria prevention. These tools are non-promotional and aim at providing the most comprehensive and up-to-date information in a way that is adapted to everyone's role.

Two of these tools deserve highlighting since they illustrate how widely different techniques can be used to convey overall similar messages to different audiences. Firstly, a "train the trainers" module is proposed to update countries' NMCP "senior trainers" on malaria management. These persons will then be responsible for cascading the information through the national public system networks so that best practices are widely used. A comprehensive training module on malaria diagnosis, treatment and prevention is delivered by African university professors through a three-day workshop organized with approximately 15 NMCP senior trainers. In addition to the technical training provided by these experts, a day is spent with a communication specialist to improve the educational techniques and teaching skills of the trainers. Together with supporting materials provided at the end of the training session, the NMCP senior trainers are thus better equipped to organize effective training sessions for their colleagues in the field.

Another initiative called "Schoolchildren against Malaria" was designed, aimed at young children approximately 10 years of age. In this programme, schoolchildren are taught about the basics of malaria, with a focus on preventative measures at the community level, and are asked by their teachers to design a theatre play on this theme.

To create some emulation, a competition is organized among schools, and the final contest for the best play is organized as a festive event. Through this approach, children not only learn about malaria but that they will also act as information relays for their families and friends. Between 2008 and 2010, a total of approximately 200,000 children were involved in this initiative in Côte d'Ivoire, Ghana and Burkina Faso. This approach, which builds on the African tradition of oral culture and theatre, will be expanded to additional countries in the coming years. Educational tools offered to countries and NGOs that want to introduce ASAQ Winthrop are not protected by patents or copyrights so that they can be used, copied and adapted as broadly as needed, according to each country's needs and priorities.

#### Expanding global partnerships

As is the case for HIV/AIDS and tuberculosis, most of the malaria drugs made available in public health systems are bought through international agencies (such as the Global Fund to fight AIDS, tuberculosis and malaria, the UNICEF, the President's Malaria Initiative, etc). With the advent of the Affordable Medicines Facility-malaria (AMFm) initiative, major adaptations to the packaging of eligible medicines are requested by the funders, to enable, on a country-by-country basis, the identification of drugs purchased through this facility [12]. Although one could see interfaces between purchasing agencies and pharmaceutical firms as merely commercial relationships, information exchanges are so intense between providers and purchasers that they often times develop into true partnerships that ensure the quality, availability and affordability of malaria drugs to patients.

## Discussion and evaluation

This paper intends to illustrate how a two-sided product development partnership initiated to develop a new anti-malarial drug has evolved to include additional partners to address the challenges posed by the launch of this new drug, its effective introduction in national programmes, and the need to ensure a steady supply. What lessons can be drawn from this experience? In early 2007, when sanofi-aventis and DND*i *launched ASAQ Winthrop, their partnership was perceived as an unlikely marriage. Nevertheless, one of the world's largest pharmaceutical companies and a not-for-profit R&D organization successfully brought their respective strengths together in order to respond to an urgent public health need. Through their efforts, the organizations managed to provide patients with a new anti-malarial and prove that public and private organizations can successfully work together to achieve a common objective.

A successful partnership requires a common desire, complementary knowledge, and shared responsibilities. The ASAQ Winthrop partnership between sanofi-aventis and DND*i *possessed all of these attributes. ASAQ Winthrop represents a good example of needs-driven innovation. While it does not represent an innovative new molecule developed from the benchtop (which often requires decades of development and a small chance of success), it represents a very significant innovation for patients in resource-poor settings. In a disease where drug resistance represents a major problem, a fixed-dose combination with a simple dosing regimen provides a clear improvement over non-fixed combination of the two separate drugs. The fixed-dose combination ensures that patients take both drugs simultaneously and in the correct proportions. The additional advantage of tablets that can be dissolved in water was specifically developed for children and it spared the need to develop a distinct pediatric formulation, which would have required additional time and funds.

The availability of a new medicine does not imply access in the field. As such, the partnership made two bold commitments. First, the product would receive no patent protection. Second, the partners set a target price of one 1 USD per treatment for adults and 0.5 USD cents for children. Before ASAQ Winthrop's introduction to the marketplace, the price for most ACTs in public markets was approximately 2.50 USD for an adult treatment. After ASAQ Winthrop's introduction, the global reference price for ACTs on public markets decreased to approximately 1 USD.

Clearly, two organizations with different *raisons d'être *will not always agree and will have different views and approaches towards achieving a common goal. This has, on more than one occasion, led to debates, and delayed decisions. The ASAQ Winthrop partnership, sanofi-aventis and DND*i *learned how to utilize their respective skills to have the greatest possible impact. For instance, sanofi-aventis provided its expertise in regulatory practices that was critical in obtaining registration in countries and prequalification by the WHO, and DND*i *stressed the importance of providing instructions on packages that patients with low literacy could understand. In addition, the two organizations engaged complementary networks of contact organizations and found that dialogue and actions were often times easier and more fruitful when the partners worked together rather than in isolation. Lastly, the launch of ASAQ Winthrop in 2007 drew wide media attention in both specialized and lay press. It is possible that the global media exposure of the "$1 malaria treatment" played a role in establishing the new reference price for anti-malarials. Both organizations now understand the importance of creative and innovative contracts between public-private partnerships, and that joining forces in a common development team can allow for programme acceleration.

Both organizations decided to extend their successful collaboration to ensure the deployment of ASAQ Winthrop and monitor its long-term safety and efficacy. This was formalized through the Risk Management Plan, that is partly supported by Medicines for Malaria Venture and by the royalties that sanofi-aventis pays to DND*i *from sales on the private markets. The lessons learned from this experience could prove invaluable in assessing future drugs' efficacy and safety in the field. Partnering with NMCPs to design and implement informational tools and techniques proved to be a very rich, and ongoing, experience. Each country has its own priorities in terms of information needs, and offering a variety of tools enables targeting specific audiences with adapted tools and techniques. While tools intended for health professionals usually only require minor country-specific adaptations, those intended for communities often require translation into local languages, as well as the adaptation of pictures and drawings, so that local populations effectively relate with the messages. Similarly, collaboration with international purchasing agencies requires adaptation to each organization's requirements and working habits, to establish long-term effective relationships. Lastly, the importance of the contribution of public and philanthropic funders at all stages of the partnership cannot be overemphasized.

Overall, the result of the work performed by all partners can be measured in the speed and extent with which this new drug has been made available to African patients. As of late 2010, ASAQ Winthrop is registered in 30 sub-Saharan African countries and in India, and, in the 3 years that have followed its prequalification by the WHO, over 80 million treatments have been purchased by governments or NGOs in 21 countries. To date, 10 clinical studies, sponsored by sanofi-aventis, DND*i *or academic teams, have been completed, involving 3432 patients and 9986 malaria episodes treated with ASAQ Winthrop, in 16 African countries, as well as in India and in Colombia (Table 2).

**Table 2:** Comparative clinical trial data with ASAQ Winthrop (as of December 2010)

Comparator drug	Country, Reference	Patients	Patients receiving ASAQ Winthrop
Non-fixed combination of aretsunate + amodiaquine	Burkina Faso [5]	Children < 5 years	375

Amodiaquine	India [SP]	Children and adults	201

Artemether-lumefantrine	Senegal, Mali, Cameroon, Madagascar [8]	Children and adults	629

	Benin [13]	Children < 10 years	90

	Liberia [14]	Children < 5 years	150

	Liberia [15]	Children and adults	496

	Senegal [16]	Children and adults	184

	Colombia [SP]	Adults	105

	Uganda [SP]	Children < 5 years	200

Artemether-lumefantrine and DHA-piperaquine	Burkina Faso, Gabon, Mozambique, Nigeria, Rwanda, Uganda, Zambia [17]		1002

Total			3432

[SP] Submitted for publication

## Conclusion

Solutions to complex problems need the different and complementary skills of various partners. The speed at which ASAQ Winthrop was adopted by public health officials and by patients in malaria-endemic countries shows that this drug fitted a need. It also highlights the efficacy of recent international efforts to bring malaria medicines where they are needed, in particular more efficient drugs procurement systems through better collaborations between endemic countries and funding bodies. In this evolving and expanding partnership, all involved have learned, and continue to learn valuable skills in how public and private organizations can collaborate effectively. This success will hopefully serve to inspire other organizations to consider the benefits of public-private partnerships.

## Abbreviations

ACT: artemisinin-based combination therapy; ASAQ Winthrop: Coarsucam^® ^/Artesunate Amodiaquine Winthrop^®^; DND*i*: Drugs for Neglected Diseases *initiative*; FACT: Fixed-Dose Artesunate-based Combination Therapies (FACT) Project; RMP: Risk Management Plan; WHO: World Health Organization; NMCP: National Malaria Control Programme; NGO: Non-Governmental Organization.

## Competing interests/Conflict of interests

FB and RS are employed by sanofi-aventis, JRK and BP are employed by the Drugs for Neglected Diseases *initiative*.

## Authors' contributions

FB wrote the initial manuscript, JRK, RS and BP made substantial contributions and helped to finalize the manuscript. All authors read and approved the final manuscript.
